# The Role of an Interdisciplinary Left-Ventricular Assist Device (LVAD) Outpatient Clinic in Long-Term Survival After Hospital Discharge: A Decade of HeartMate III Experience in a Non-Transplant Center

**DOI:** 10.3390/biomedicines13081795

**Published:** 2025-07-22

**Authors:** Christoph Salewski, Rodrigo Sandoval Boburg, Spiros Marinos, Isabelle Doll, Christian Schlensak, Attila Nemeth, Medhat Radwan

**Affiliations:** Department of Thoracic and Cardiovascular Surgery, Tübingen University Hospital, 72076 Tübingen, Germanyisabelle.doll@med.uni-tuebingen.de (I.D.); medhat.radwan@med.uni-tuebingen.de (M.R.)

**Keywords:** heart failure, LVAD, outpatient clinic

## Abstract

Background: In advanced heart failure patients implanted with a fully magnetically levitated HeartMate 3 (HM3) Abbott left ventricular assist device (LVAD), it is unknown how the role of the LVAD outpatient clinic may affect the long-term survival after hospital discharge. Our objective is to share our standardized protocol for outpatient care, to describe the role of the LVAD outpatient clinic in postoperative long-term care after LVAD implantation, and to report survival. Methods: We retrospectively reviewed all patients implanted with HM3 LVAD in our institute between September 2015 and January 2025. Patients who received HeartWare Ventricular Assist Device (HVAD) and HeartMate 2 LVAD devices were excluded from our study, to ensure a homogenous cohort focusing on the latest and the only currently used LVAD device generation. We included a total of 48 patients. After LVAD patients are discharged from our center, they are followed in our outpatient clinic in 3-month intervals. During visits, bloodwork, EKG, wound inspection, and echocardiography are performed in addition to LVAD analysis. The role of the outpatient clinic is to detect early signs of deterioration or problems and act accordingly to prevent serious complications. **Results**: Thirty-three patients (68.7%) are still alive in 2025; two patients (4.2%) had a successful heart transplantation; and thirty-one patients (64.5%) are still on LVAD support. There were 210 total patient years of support. The mean time on device is 4.4 years. During the follow-up period we noticed 15 deaths (31.3%). Notably, there was no technical device-related death. Kaplan–Meier analysis estimated an overall survival rate of 97.9%, 92.8%, 83.7%, and 51.1% at 1, 2, 4, and 8 years, respectively. **Conclusion**: Strict control of patients after discharge in an outpatient clinic is essential for the long-term survival of these patients. A well-structured outpatient program is of utter importance to avoid LVAD-related complications and should be a cornerstone for the treatment, especially in non-transplant centers.

## 1. Introduction

Left ventricular assist devices (LVADs) have become a cornerstone in prolonging survival and improving quality of life for patients with advanced heart failure refractory to medical therapy [1]. They even have better survival, especially on the HM 3 [2,3].

This success can be attributed to the newest centrifugal pumps, which are characterized by reduced dimensions and higher biocompatibility. In particular, the HM 3 represents a technological paradigm based on its design. With the hallmarks of a fully magnetically levitated motor (Full MagLev) with no mechanical contact points, larger gaps, sintered surfaces, artificial pulse, and modular driveline, HeartMate 3 (HM3) significantly improved patients’ outcomes compared with previous generations of LVADs [4].

In Germany, the demand for heart transplantations (HTX) exceeds the available organ number by far (approx. demand of 1000 hearts and 300 available per year). To maintain HTX outcome quality, transplantation authorization was given to specialized transplant centers only. However, other larger (university) medical centers contribute to the treatment of the demand gap by fostering VAD programs as well. We would like to contribute our data to the body of knowledge as a low-volume non-transplant center. After discharge, patients require specialized care to prevent severe complications and ensure long-term survival. In our center, after discharge, follow-up is performed in our outpatient clinic, which patients have to visit at least once every 3 months. Data on the role of the specialized LVAD outpatient clinics (OC) is limited. A PubMed search with the terms “LVAD outpatient clinic” returns 27 hits. Most of which illuminate only one aspect of the LVAD patient treatment (e.g., aortic valve opening [5,6], bleeding [7], infections [8], community support [9], rehabilitation, economics [10], QoL [11], anticoagulation [12,13,14], HU listing [15], and renal failure [16]) but do not provide a protocol on how to build a program. A more specific search led to the program suggested by Sokos et al. [17]. They suggest a program for remote non-transplant centers.

### Scientific Novelty and Purpose of the Study

This study addresses the importance of a standardized outpatient care protocol and the integral role of an interdisciplinary LVAD outpatient clinic in the long-term management of HM3 LVAD recipients at a non-transplant center.

Furthermore, by exclusively focusing on the HeartMate 3 device, we provide a homogenous dataset that minimizes confounding variables associated with different device generations.

Our study aims to describe a well-defined outpatient protocol, thereby serving as a model for other non-transplant centers. We want to add our data to the body of knowledge. By proactively monitoring, detecting complications early, and optimizing management strategies within our structured outpatient clinic, we aim to contribute to long-term survival rates even as a low-volume non-transplant center.

## 2. Materials and Methods

We retrospectively analyzed 48 consecutive patients who were successfully discharged after LVAD implantation of HM 3 in our center between September 2015 and January of 2025.

Exclusion Criteria for Other Devices: It is important to clarify the rationale behind the exclusion of patients implanted with HeartWare Ventricular Assist Device (HVAD) and HeartMate 2 (HM2) LVADs. This decision was made to ensure the homogeneity of our study cohort, allowing for a focused evaluation of outcomes specifically associated with the latest generation HM3 device. The HM3, with its unique design features such as full magnetic levitation and an artificial pulse, has demonstrated distinct clinical outcomes and complication profiles compared to previous generations [4]. Therefore, including patients with older devices would introduce confounding variables that could obscure the specific impact of our outpatient protocol on HM3 recipients.

We collected preoperative data from the patients, such as gender, age at implantation, indication for implantation, duration of device therapy, and hospital admissions after discharge.

All the patients who undergo an LVAD implantation in our institution are commenced on a standardized outpatient protocol.

- The role of the specialized cardiac surgeon in the LVAD outpatient clinic:

The cardiac surgeon leads the team in postoperative management and works closely with the multidisciplinary team to ensure quality care is provided. The role of the cardiac surgeon also varies depending on the level of care required. In our center, outpatient care for LVAD patients is primarily the responsibility of the cardiac surgeons; admissions are also regulated and organized through the specialized LVAD team and occur mainly in the cardiac surgery department. Every week in the LVAD outpatient clinic, a member of the team carries out a standardized clinical examination of the patients: a transthoracic echocardiography exam addresses heart failure symptoms and potential complications (right heart failure, ventricular thrombosis, infections of the subcutaneous trajectory of the driveline); they discuss with the LVAD coordinator and cardiologist for decision-making; they do a driveline and wound examination; they adjust medications, such as vitamin K antagonists; and monitor the laboratory findings. Subsequently, all the findings are discussed with the patients, further recommendations are made, changes in therapeutic medications and the time of the next visit are planned.

- The role of the specialized cardiologist in the LVAD outpatient clinic:

The specialized cardiologist co-leads the LVAD program in our center. In the LVAD outpatient clinic they are responsible for the adjustment of therapeutic heart failure medications, monitoring heart failure symptoms and potential complications (right heart failure, stroke, infections), adjustment and control of pacemakers and ICDs, and coordination with the LVAD coordinator and surgeon for decision-making.

- The role of the specialized LVAD coordinator in the LVAD outpatient clinic:

In our center the LVAD coordinator plays a crucial role in the primary care provided for all LVAD patients. His/her qualification is a nurse/specialized heart failure nurse. The coordinator organizes early phone calls post discharge and coordinates post-discharge clinic visits (every patient visits the LVAD outpatient clinic at least once in three months, and more visits are also organized when indicated). In the outpatient clinic our LVAD coordinator ensures proper device function, checks for alarms and mechanical issues, and routinely examines and provides systemic driveline wound care. Generally, our LVAD coordinator also guides other medical staff on specific LVAD protocols and acts as a bridge between the hospital LVAD team and other medical care personnel.

- The role of the specialized technical support in the LVAD outpatient clinic:

All LVAD alarms and technical issues are covered through the HM3 technical support via telephone calls and device protocol scanning. In case of alarms or sudden deterioration, the LVAD log file can be retrieved from the controller and sent to the technical support. Immediate help can be sought around the clock, so pump-related reasons for the patients’ primary complaint can be ruled out or confirmed.

**Figure 1:**
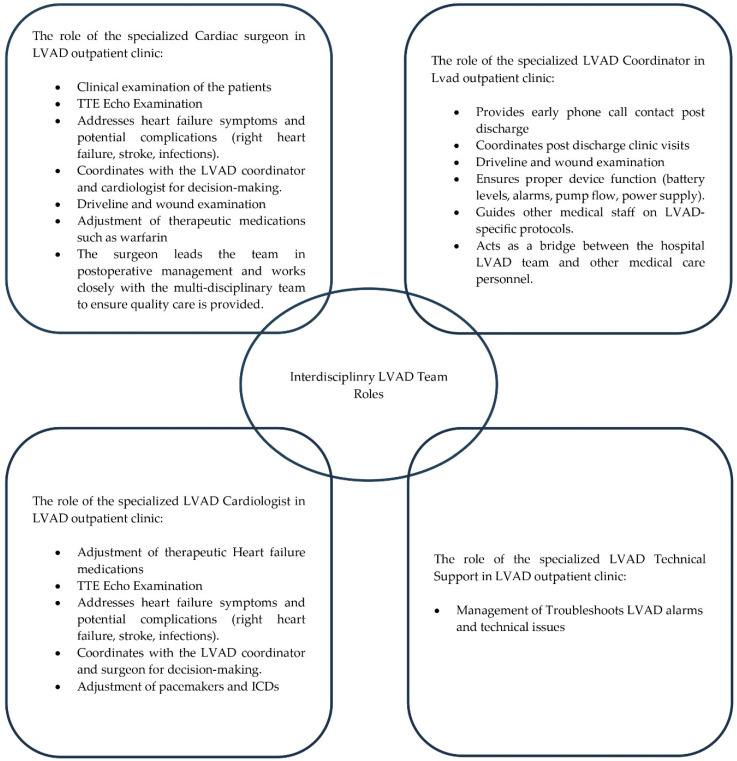
Summarizes our standardized protocol in the LVAD outpatient clinic. Inspired by Ref. [17].

The protocol is summarized in Figure 1.

### 2.1. Endpoints

The primary endpoint was evaluating the role of the LVAD outpatient clinic to identify patients with a high risk for complications.

Secondary endpoints included fatal LVAD-related complications and causes of mortality after hospital discharge. Adverse events were defined according to the latest ISHLT definition of adverse events for trials and registries of mechanical circulatory support [18].

### 2.2. Statistical Analysis

Statistical analyses were performed using the SSPS 28.0 (IBM Corporation, Armonk, NY, USA) software. Normal distribution was checked using the Kolmogorov–Smirnov test. Continuous variables are reported as means and standard deviations if they fulfill the criteria of a normal distribution; otherwise, median and interquartile ranges are reported. The ethics committee of the University of Tübingen, Germany, approved the study with the project No. 194/2020BO2. Due to the retrospective nature of the study, informed consent was not necessary.

## 3. Results

Forty of the patients in this cohort were men (83.3%), with a mean age of 56.95 ± 10.1 years at implantation. The most common indication for LVAD implantation was ischemic cardiomyopathy in 24 patients (50%). Thirty-three patients underwent LVAD implantation as bridge-to-transplantation (68.75%), three as bridge-to-decision (6.25%), one as bridge-to-recovery (2.1%), and eleven as destination therapy (22.92%). At the time of LVAD implantation, most patients (60.7%) were INTERMACS (Interagency registry for mechanically assisted circulatory support) profile 2, followed by profile 3. Regarding pre-implantation right ventricular function, 68.7% of patients had a normal or mildly impaired function. Table 1 shows the demographic characteristics of our patients. Forty-eight (86%) patients were discharged after HM3 implantation from our center.

**Figure 2:**
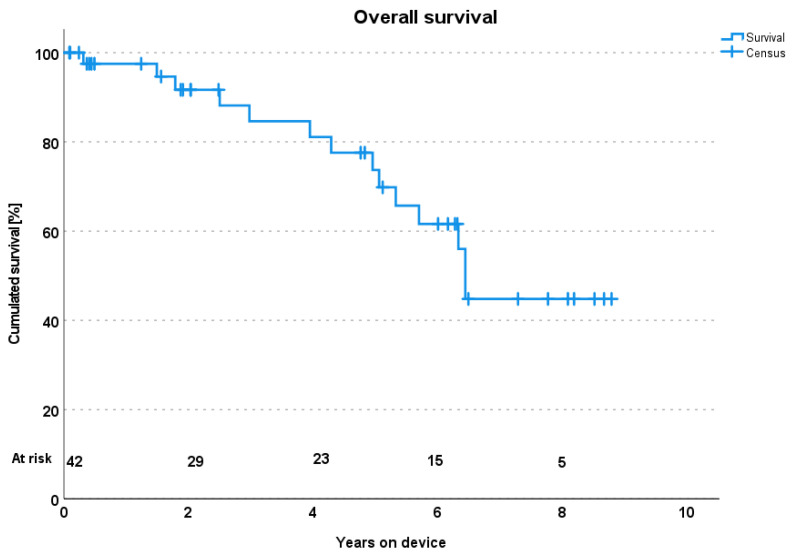
Shows Kaplan–Maier curves and estimated overall survivals.

There were 210.34 total patient years of support among the discharged patients. The mean time on the device was 4.4 years and ongoing. There were 15 deaths (31.3%) during the follow-up period. Five patients due to sepsis (10.4%), three patients due to carcinoma (6.2%), three patients due to right heart failure (6.2%), two patients due to intracranial bleeding (4.1%), one patient due to severe aortic regurgitation III (2%), and one patient due to unknown cause (2%). None of the deaths were device related. The estimated survival rate was 97.9%, 92.8%, 83.7%, and 51.1% at 1, 2, 4, and 8 years, respectively. Figure 2 shows Kaplan–Maier curves and Table 2 shows the causes of death after hospital discharge in our center.

During follow-up, there was no need for HM3 replacement. The last reported EQ-5D-5L questionnaire in 30 patients regarding quality of life was 68.5 ± 17.7%. The 30 patients reported to have 5.7 ± 4.9 out of 27 possible points regarding difficulties in everyday life in the PHQ-9 questionnaire.

Hospital readmissions from our outpatient clinic were mainly due to driveline infections in four patients (8.3%), followed by bleeding complications in three patients (6.2%). Two (4.9%) of the patients who suffered a driveline infection underwent surgical relocation; the other patients were treated conservatively (Table 3). One patient (2.4%) suffered spontaneous thoracic bleeding, another gastrointestinal bleeding, and one had postoperative bleeding after surgical debridement of a driveline infection. None of these complications were lethal. There were no thrombo-embolic events reported in this cohort.

## 4. Discussion

Continuous Flow LVAD (HM 3) is associated with an excellent hospital survival rate in patients with end-stage heart failure; discharge rates as high as 90% have been reported [4,19]. We reported a discharge rate of 85.4%.

This retrospective study emphasizes the critical role of a structured, interdisciplinary LVAD outpatient clinic in ensuring long-term survival and minimizing complications after HeartMate 3 (HM3) implantation in a non-transplant center.

The cardiac surgeon leads the team in postoperative management and works closely with the multidisciplinary team to ensure quality care is provided.

Finally, comparing the 63.3% surviving patients of the primary implant ELEVATE group with the 56.3% including the anonymous cases of the ELEVATE registry with our 73% after five years and 51% after 8 years (Figure 2) is the best we can offer. We included the relevant KM diagrams below.

A. **Interpretation of Results and Contribution of the Outpatient Clinic:** The observed 8-year survival rate of 51.1% is particularly noteworthy and compares favorably with historical data for LVAD patients from the ELEVATE registry [20]. See part B. Also, the relatively low rate of rehospitalization due to driveline infections (8.3%) and bleeding events (6.2%) further reflects the clinic’s role in preventive care. Proactive monitoring in the form of regular bloodwork, wound inspection, echocardiography, and LVAD analysis enables early detection of subtle changes, and prompt investigation and management of emerging issues prevent escalation to severe complications. We also noticed that comprehensive oversight contributes to improved patient adherence and early symptom recognition by patients themselves.
B. **Comparison with the Existing Literature and Other Clinic Models**: Our survival rates align with those reported in major LVAD registries and clinical trials for HM3 devices [4,20,21]. The ELEVATE study collected data on patients treated with HeartMate 3 after the CE mark was approved for this medical device in 2015. A total of 540 patients received an HM 3, and 26 centers contributed cases. Of the 540, 463 patients form the primary LVAD implant group, 19 received a pump exchange, and in 58 only anonymized data was collected. In the primary implant cohort (460), survival was 63.3% after five years. In our study, 73% survived 5 years and 51% survived 8 years. One patient was transplanted. We censored the transplanted patient from the count because HTX has its own risks and benefits. We do not count the patient as a contributor to survival past his explantation of the HM3. We made use of the Kaplan–Meier survival estimator. The absence of technical device-related deaths is a testament to both the advancements in HM3 technology and, crucially, the proactive monitoring and timely interventions facilitated by our outpatient clinic. The role of specialized outpatient follow-up in improving outcomes has been highlighted in several reports. For instance, Sokos et al. emphasized that multidisciplinary heart failure clinics contribute significantly to improved quality of care and reduced hospital readmissions [17]. Bosch et al. investigated healthcare consumption from an economical aspect [10]. As their number of LVAD implantations rose, so did the number of LVAD patients presenting to the LVAD outpatient clinic. This is logical. They filed a detailed report on the number of implantations, total number of patients with an LVAD, length of stay, visits to the outpatient clinic, visits to the emergency department, and readmissions.
  Despite the high burden of comorbidities and long-term support, patients in our cohort reported a mean EQ-5D-5L score of 68.5%, which aligns with prior studies showing sustained health-related quality of life in long-term LVAD recipients.
  While direct comparisons are challenging due to variations in patient populations, study designs, and follow-up durations, our data strongly support the notion that a well-structured outpatient program can achieve excellent long-term results.
  Regarding the organization of outpatient monitoring in other clinics, a review of the literature reveals a spectrum of approaches. Many centers, particularly those with high LVAD volumes, have adopted multidisciplinary clinic models similar to ours, recognizing the complex needs of this patient population [22,23]. Schaeffer et al. implemented an LVAD program and described how they follow up with their patients [24]. Monthly follow-ups by the LVAD coordinator nurse for the first 6 months, then every 3 months. Regular echo by a cardiologist and a 24/7/365 emergency direct phone chain. Creating an ICU and peripheral ward to the LVAD patients’ needs and irrespective of the reason for admission. Consultation of the VAD patient by a cardio-anesthetist and a perfusionist even for planned non-cardiac surgery. Monthly meeting of all LVAD actors for patient evaluation. However, they derived their program from Bad Oeynhausen, Germany, one of the world’s largest LVAD centers, and their program is close to ours.
  However, variations exist in the composition of the team, frequency of follow-up, and the specific protocols employed. We think that the key in our approach, which we believe contributes to our favorable outcomes, lies in the highly standardized and rigorously enforced three-month follow-up intervals, coupled with the immediate availability of a dedicated, on-site interdisciplinary team for urgent issues. This proactive and accessible model allows for rapid response to emerging complications, distinguishing it from less integrated or less frequent follow-up paradigms. While other clinics undoubtedly employ their own effective protocols, the strength of our model lies in its comprehensive, integrated, and consistently applied nature, which is particularly crucial in a non-transplant center where long-term device support is often the primary goal.
C. **Clinical Implications:** The findings of this study carry significant clinical implications; they reinforce the imperative for all centers implanting LVADs, especially non-transplant centers, to establish and maintain robust, interdisciplinary outpatient clinics. Such clinics do not merely follow up but are active centers for complication prevention, early detection, and comprehensive patient education.

### Limitations

The retrospective and single-center nature of this study are its major limitations; in order to better understand the role of outpatient care and how this influences the outcomes of patients with HM3, bigger and prospective studies are necessary. Moreover, although we describe a structured follow-up protocol, individual adherence and psychosocial factors were not quantitatively assessed. Future prospective, multicenter studies are warranted to confirm these findings and to evaluate cost-effectiveness and patient-reported outcomes across different healthcare systems. We also acknowledge that a significant limitation of this study is the absence of a concurrent control group. This makes it challenging to definitively attribute the observed beneficial effects on long-term survival solely to the work of our interdisciplinary LVAD outpatient clinic. Future research could explore comparative effectiveness studies across different outpatient clinic models or utilize propensity score matching to compare outcomes with historical cohorts or external registries, thereby strengthening the evidence base.

## 5. Conclusions

A strict follow-up in the outpatient clinic after HM 3 implantation helps detect and prevent LVAD-related complications and patient deterioration. Additionally, the LVAD outpatient clinic shows high long-term survival rates in a non-transplant center.

## Figures and Tables

**Table 1:** Patient demographic, indication, and therapy objective.

Parameter	*n* = 48
Gender (m)	40 (83.3%)
Age (y)	61.4 ± 10.4
**Indication for LVAD Implantation**	
Ischemic cardiomyopathy	25 (52.1%)
Dilatative cardiomyopathy	20 (39.6%)
Myocarditis	4 (8.3%)
**Preoperative INTERMACS Profiles**	
2	29 (60.7%)
3	13 (27.08%)
4	5 (10.4%)
5	1 (2.08%)
**Preoperative Echocardiographic Right Ventricular Function**	
Normal	18 (37.5%)
Mildly impaired	15 (31.2%)
Moderately impaired	12 (25%)
Severely impaired	3 (6.2%)
Bridge-to-transplant	33 (68.75%)
Bridge-to-decision	3 (6.25%)
Bridge-to-recovery	1 (2.1%)
Destination therapy	11 (22.92%)

INTERMACS: Interagency registry for mechanically assisted circulatory support.

**Table 2:** Causes of mortality after hospital discharge.

	*n* = 41
Sepsis	5 (10.4%)
Carcinoma	3 (6.2%)
Right ventricular failure	3 (6.2%)
Intracranial bleeding	2 (4.1%)
Severe aortic regurgitation	1 (2%)
Unknown	1 (2%)

**Table 3:** Shows the causes of readmissions from the LVAD outpatient clinic during the follow up period.

Hospital Readmissions	
**Cause**	***n* = 48**
Driveline infection	4 (8.3%)
Bleeding complications	3 (6.2%)
Heart transplantation	1 (2%)

## Data Availability

The raw data supporting the conclusions of this article will be made available by the authors on request.
